# Integrating sensory analysis and voltammetry to explore oxidative susceptibility in red wines^☆^

**DOI:** 10.1016/j.fochx.2026.104163

**Published:** 2026-07-02

**Authors:** Mónica Bueno, María-Pilar Sáenz-Navajas, Cristina Peña, Ignacio Arias, Carolina Castillo, Purificación Fernández-Zurbano, Arancha De la Fuente-Blanco, Chelo Ferreira, Ana Escudero, Vicente Ferreira

**Affiliations:** a Laboratorio de Análisis del Aroma y Enología (LAAE), Department of Analytical Chemistry, Universidad de Zaragoza, Instituto Agroalimentario de Aragón (IA2) (UNIZAR-CITA), c/Pedro Cerbuna 12, 50009 Zaragoza, Spain; b Instituto de Ciencias de la Vid y del Vino (ICVV) (UR-CSIC-GR), Finca La Grajera, 26007 Logroño, La Rioja, Spain; c Instituto Universitario de Matemáticas y Aplicaciones (IUMA-–UNIZAR), Universidad de Zaragoza, c/ Pedro Cerbuna 12, 50009 Zaragoza, Spain

**Keywords:** Wine oxidation, Voltammetry, Phenolic compounds, Oxidative susceptibility, Sensory analysis

## Abstract

Wine longevity is a key determinant of quality in premium wines, yet reliable tools to predict susceptibility to deterioration remain limited. This study evaluated longevity using two accelerated ageing models: controlled oxidation (COx) and anoxic thermal treatment (ATT). Twelve commercial red wines were subjected to both treatments and assessed by sensory analysis, tannin activity and voltammetric profiling. COx induced clear sensory deterioration, including loss of fruity aromas, development of oxidative notes, and increased bitterness and astringency, whereas ATT produced minor changes. Oxidation was also associated with increased tannin activity, consistent with oxidative modifications of the phenolic matrix. Voltammetric analysis revealed treatment-dependent changes in key redox-active regions. A nested PCA–LDA approach classified wines according to oxidation susceptibility with 83.3% accuracy. These findings demonstrate that electrochemical profiling captures meaningful information on wine redox behaviour and highlight its potential as a rapid screening tool to support ageing, storage and quality management decisions.

## Highlights

• Controlled oxidation induced greater sensory deterioration than thermal ageing.
• Oxidation increased tannin activity and altered redox-active regions.
• Nested PCA–LDA classified oxidation susceptibility with 83.3% accuracy.
• Key electrochemical regions were associated with oxidation susceptibility.
• Voltammetry captured redox features linked to wine oxidative behaviour.

## 1 Introduction

Wine quality is closely intertwined with wine longevity, since the ability of a wine to evolve positively over time is regarded as a hallmark of premium products. Picard, Tempere, de Revel, and Marchand (2015) emphasise that the tendency of wine to improve with age is one of its most distinctive attributes, reflecting the gradual development of complexity, harmony, and aromatic depth during bottle maturation. Conversely, premature oxidative degradation or the emergence of faulty ageing notes represents a clear decline in wine quality, illustrating that longevity is not merely a temporal concept but a fundamental expression of chemical and sensory stability (Oliveira, Ferreira, De Freitas, & Silva, 2011).

Climate change further exacerbates this challenge: rising temperatures disrupt grape phenolic maturity, increase pH, and elevate sugar concentrations (Venios, Korkas, Nisiotou, & Banilas, 2020). These effects alter the balance and accumulation of grape secondary metabolites such as polyphenols and aroma precursors, leading to aromatic deviations such as atypical ageing notes, prune-like aromas, and green or herbaceous characters (Pons et al., 2017) as well as negative mouthfeel properties (Sáenz-Navajas et al., 2018). Alongside these flavour changes, amino acids or the thiol-containing tripeptide glutathione (GSH) play an essential protective role by scavenging quinones formed during phenolic oxidation (Nikolantonaki & Waterhouse, 2012), thereby preserving aroma precursors and limiting oxidative deterioration (Aragón-Capone, Bueno, Marzo-Méndez, Escudero, & Ferreira, 2025). Because GSH or amino acid levels also depend on vineyard conditions, climate-driven metabolic shifts directly affect both the sensory quality directly impacting wine shelf-life and undermine the capacity of wines to develop favourably after bottling (Pons et al., 2017). Thus, being able to predict a wine's capacity to age gracefully is essential for preserving its long-term quality.

Recent critical reviews have questioned whether chemical analysis alone can reliably predict wine ageing capacity. Waterhouse and Miao (2021) examined a wide range of potential chemical indicators–including phenolic composition, antioxidant capacity, and redox-related parameters–and concluded that, while individual markers provide valuable insights into wine susceptibility to oxidation, no single chemical parameter is sufficient to robustly predict ageing performance. Instead, they advocate for integrative approaches combining chemical fingerprints, controlled ageing conditions, and sensory outcomes. This perspective reinforces the need for multivariate, mechanism-oriented tools capable of anticipating deterioration risks before sensory defects become perceptible.

Recent experimental evidence confirms that oxidation is not merely a process of compositional loss, but also one of molecular formation leading to undesirable aroma attributes. A recent study on Spanish red wines subjected to accelerated oxidation revealed profound alterations in the aromatic profile through sensory analysis and GC-olfactometry, highlighting the formation of Strecker aldehydes, acetals, and sotolon as key markers of oxidative deterioration (Aragón-Capone, De-La-Fuente-Blanco, Sáenz-Navajas, Ferreira, & Bueno, 2025). Complementary studies comparing oxidative and anoxic thermal storage demonstrated that both conditions induce qualitatively similar ageing-related changes, although oxidation produces substantially more intense effects (Aragón-Capone, Bueno, & Ferreira, 2026). Time-dependent reactions such as hydrolysis of glycosidic precursors, esterification equilibria, and molecular rearrangements occurred under both conditions, whereas acetalisation and Strecker degradation were markedly enhanced by oxygen exposure. Additional oxidation-specific effects included terpenol and methionol oxidation, degradation of phenolic acids into vanillin derivatives, and losses of non-polar aroma compounds. Importantly, SO₂ modulates these processes through quinone reduction and aldehyde binding, contributing to oxidative stability. Overall, the accumulation of free acetaldehyde, diethyl acetal, and Strecker aldehydes emerged as the most sensory-relevant consequences of oxidative ageing.

Recent metabolomic studies (Razafindrabenja et al., 2026) further support the central role of oxygen exposure in wine longevity through its impact on polyphenol evolution. Oxygen promotes structural modifications of condensed tannins, including A-type linkages, sulphonation, tannin-anthocyanin condensations, pyranoanthocyanin adducts, polycondensation products or tannin-aroma interactions involving mercaptans such as 4-mercapto-4-methylpentan-2-one or 3-mercaptohexanol (Suc, Rigou, & Mouls, 2021). Wines exposed to lower oxygen transfer rates showed more stable tannin profiles and lower levels of oxidation-derived markers than wines under higher oxygen exposure. These findings reinforce the relevance of redox-active phenolic systems as markers of oxidative stability and wine longevity. Within this scientific framework, substantial gaps remain in the prediction of wine longevity, particularly regarding the combined roles of polyphenols, aroma compounds, and their precursors. In this study, wine longevity is investigated using two complementary accelerated ageing models:

(i) controlled oxidation (COx), designed to generate accelerated oxidative stress conditions capable of differentiating wines according to their oxidation susceptibility and promoting quinone formation, Strecker degradation, pigment evolution, tannin polymerisation, and loss of varietal mercaptans.
(ii) thermal treatment under anoxia (ATT), which reproduces temperature-driven ageing processes occurring in sealed environments and promotes reactions such as hydrolysis of glycosidic precursors, esterification equilibria, and molecular rearrangements.

To obtain a rapid assessment of wine susceptibility to deterioration under COx and ATT conditions, voltammetric measurements were evaluated to determine whether initial electrochemical signatures encode information related to ageing behaviour. This approach is consistent with emerging sensometabolomic perspectives (Sáenz-Navajas, Iosifidis, Gonzalez-Hernandez, Fernández-Zurbano, & Valentin, 2023), which aim to link chemical fingerprints with sensory evolution.

Based on this context, the objectives of the present study are to:

(i) evaluate wine evolution under two mechanistically differentiated accelerated ageing conditions, namely controlled oxidative stress (COx) and anoxic thermal ageing (ATT);
(ii) investigate the relationship between electrochemical behaviour, phenolic reactivity, and sensory oxidation susceptibility;
(iii) assess the potential of voltammetric approach as rapid indicator of wine oxidative stability and longevity.

The hypotheses tested are:

**H1.** Wines subjected to accelerated oxidative (COx) and anoxic thermal ageing (ATT) conditions will exhibit different sensory and electrochemical deterioration patterns depending on their intrinsic oxidation susceptibility.

**H2.** Initial voltammetric profiles may contain electrochemical signatures associated with differences in wine oxidation susceptibility.

## 2 Materials and methods

### 2.1 Wine samples

Twelve commercial Spanish red wines were selected to cover different ageing categories, production styles, and expected oxidation and reduction susceptibilities. These included both young wines (i.e., wines with maximum shelf-life of 2 years) and barrel-aged wines designed for medium-term evolution (i.e., crianza wines with a maximum shelf-life of 6 years). Wine selection was performed following a preliminary sensory screening to ensure variability in fruity and woody intensity, and mouthfeel attributes, and ensure absence of aromatic defaults (reduction, oxidation, animal, TCA). Therefore, a bench test was performed with 25 commercial wines and 5 experimenters. The initial list of the 25 wines, ranging in price from 3 to 15€ per bottle, was generated with the premises that the sample set shows a great chemical variability, thus Spanish wines from different wine regions and varieties were firstly selected (list of the 25 wines is in Table S1 and conventional parameters for the 12 selected samples in Table S2 of Supplementary Material).

Nine bottles of each of the 12 selected wines were submitted to three experimental treatments, yielding 36 different final samples (12 wines × 3 conditions), which constitute the central experiment.

### 2.2 Preliminary experiments

Preliminary sensory studies were conducted with three and four wines, respectively, to optimise the conditions of both the anoxic thermal treatment (ATT) and controlled oxidation (COx) treatments applied in the central experiment. This preliminary step allowed the identification of treatment conditions capable of inducing measurable sensory deviations while preserving inter-wine variability. Accordingly, wines were subjected to ATT conditions (strict anoxia at 35 °C) for up to 14 weeks and to COx conditions combining different temperatures (6–50 °C) and oxygen doses (0–65 mg/L above the stoichiometric amount required to oxidise total SO₂) for 10 weeks. The ATT and COx conditions evaluated to generate the experimental samples are described below. The resulting samples were analysed by descriptive sensory analysis, whose methodological details are provided in Section 2.3.1.2. For each wine and condition, the sensory data were analysed by analysis of variance (ANOVA), considering judge as random factor and treatment as a fixed factor.

#### 2.2.1 Thermal stability tests: ATT

Three wines were incubated at 35 °C under strict anoxia for 14, 10, 5, 2 and 0 weeks to study the kinetics of fruit degradation, reduction markers, and changes in astringency over time. After incubation, bottles were equilibrated at 6 °C before sensory screening analysis. Results showed that 10 weeks were sufficient to induce measurable thermal degradation in red wines at 35 °C (see Preliminary Experiments Fig. S1 in Supplementary Material).

#### 2.2.2 Controlled oxidation tests: COx

Four wines were subjected to oxidative treatments for 10 weeks, combining temperature (6, 22, 35 and 50 °C) and initial oxygen doses consisting of either no added oxygen (0 mg/L of oxygen) or 35, 50, 65 mg/L over the stoichiometric amount to oxidise total SO_2_. Treatments followed a previously described procedure (Marrufo-Curtido, Carrascón, Bueno, Ferreira, & Escudero, 2018), adapted here to 750-mL bottles. The headspace volume required to achieve each nominal oxygen level was calculated considering wine density, oxygen solubility, bottle volume and SO₂ stoichiometric consumption. Wine-In-Tubes equipped with integrated Pst3 oxygen sensors were prepared in duplicate to monitor O₂ consumption kinetics using a Fibox 3 LCD-trace analyser, ensuring that the supplied oxygen was consumed within the 10-week period. The condition combining 35 mg/L of O₂ above the stoichiometric SO_2_ requirement and 35 °C was selected for the central experiment, as it produced detectable oxidative sensory deviations without inducing atypical spoilage (Aragón-Capone, Bueno, et al., 2025). Detailed sensory profiles of the resulting samples are provided in Preliminary Experiments section Figs. S2 and S3 of the Supplementary Material.

### 2.3 Central experiment

Each of the 12 selected wines were incubated for 10 weeks and assigned to three final conditions: (i) Control (C) (anoxia, 6 °C): bottles stored under refrigerated anoxia to simulate ideal preservation conditions and used as a proxy for the initial state of the wines before treatments; (ii) Anoxic thermal treatment (ATT) (anoxia, 35 °C): simulating accelerated shelf-life ageing under oxygen-free conditions; (iii) Controlled oxidation treatment (COx) (35 °C + 35 mg/L O₂ above the stoichiometric amount required to oxidise total SO_2_), where bottles were prepared with a precisely defined air headspace to provide the desired oxygen dose while accounting for the stoichiometric oxygen consumption by SO₂.

Nine bottles were used for each of the 12 selected wines (3 bottles for C, 3 bottles for ATT, and 3 bottles for COx). All wines were resealed using DIAM Bouchage DIAM 30 cork closures under controlled conditions to standardise oxygen ingress and eliminate variability from original closures. According to commercial specifications, these closures contain an initial internal oxygen content of approximately 0.8 mg O₂, which is typically released over about 6 months, plus an additional permeation rate of 0.3 mg O₂ per bottle per year under standard conditions. To minimise the contribution of the initial oxygen contained in the closures, the corks were kept for at least 48 h in an anoxic chamber prior to use to allow oxygen desorption. Subsequently, the closures were stored in double-sealed bags with activated carbon placed between layers during transport to the bottling facility to further reduce oxygen exposure. After bottling, an oxygen-impermeable seal was applied to the upper surface of the cork using a two-component rapid-curing epoxy adhesive (Araldite® Rapid, CEYS, Spain) to prevent oxygen ingress at the closure interface.

The six bottles assigned to C and ATT conditions were opened and immediately sealed using a commercial vacuum bottling line (Bodegas y Viñedos Ilurce, La Rioja) operating at −0.25 bar to ensure bottle oxygen removal prior to closure. Before recorking, a small volume of wine (20 mL) was removed. The corks and the removed wine were evaluated by orthonasal sensory inspection to detect possible initial defects, including the presence of 2,4,6-trichloroanisole (TCA), and to prevent wine overflow due to thermal expansion. Contaminated bottles were discarded.

The remaining three bottles per wine were used for the controlled oxidation treatment (COx). For these samples, the volume of wine was reduced to generate the headspace required to introduce the desired amount of oxygen, following the methodology described in Section 2.2.2. The extracted wine was also evaluated to rule out initial faults. These bottles were recorked with the same DIAM 30 closures without applying vacuum.

Incubations began between 21–22 March 2024 and ended 29–30 May 2024. All bottles were transferred to 4 °C storage after the 10 weeks and all analyses were carried out within three weeks after the end of the incubation.

#### 2.3.1 Sensory characterisation

The study protocol was approved by the CSIC Ethics Committee (internal code: 166/2024) and conducted in accordance with applicable ethical standards for research involving human participants. Participation was voluntary, and no personally identifiable information was collected. Participants were first informed about the procedures of the sensory study, after which written informed consent was obtained from all volunteers prior to their participation.

Samples were submitted to projective mapping by a panel of experts and descriptive analysis by a trained panel. In both tasks, 20-mL per sample were served in black ISO wine glasses (ISO 3591:1992) with Petri dish covers. All samples were coded with random three-digit numbers and presented in a different order for each participant following a Williams Latin square. Water and pectin (1 g L^−1^) solution were employed as rising agents. Sessions were conducted in individual booths under controlled white lighting and temperature, using RedJade software for data capture for descriptive analysis and paper ballot for projective mapping.

##### 2.3.1.1 Projective mapping

*Participants:* A total of 12 wine experts took part in the experiment, comprising nine women and three men. The participants had all accumulated extensive experience in the production of red wine in Rioja area, ranging from a minimum of five to a maximum of 35 years, with an average experience of 16 years. The age distribution of the subjects was as follows: six individuals were in the 30–39 age range, and two individuals in each of the 20–29, 40–49 and 50–59 age ranges.

*Sample evaluation:* Experts participated in two sessions in the same day separated by one hour. In each session, participants were presented with 24 samples simultaneously (12 control samples +12 treated samples: COx or ATT). A counterbalanced design was implemented: half of the participants were presented with COx and control samples in Session 1 and ATT and control samples in Session 2, whereas the other half received the treatments in the reverse order. Participants were asked to arrange the 24 samples on the tablecloth according to their sensory similarity, so that samples that are more similar were placed closer together and more different samples further apart. Participants were then asked to score each sample for their perceived quality on a scale of 0 to 10 (0 poor; 2.5: low quality; 5 average quality: 7.5: high quality; 10: exceptional) and their REDOX state (score from −5: strongly reduced to +5: strongly oxidised; 0 was defined as ‘neither oxidised nor reduced’).

*Data analysis:* Initially, the coordinates (X, Y) for each sample and participant were recorded, thereby generating a matrix for each test (control + COx samples or control + ATT samples). In the subsequent stage of the analysis, a generalised procluster analysis (GPA) was calculated. In this analysis, the coordinates (x, y) given by each judge to each wine were considered a matrix. For quality and REDOX scores a two-way ANOVA considering participants as random and the treatment as fixed factor was calculated in order to evaluate the effect of the treatment.

##### 2.3.1.2 Descriptive analysis

*Participants:* A trained panel of 12 assessors, all researchers from LAAE and ICVV with extensive experience in wine descriptive analysis, participated following UNE-EN ISO 8586 guidelines. Panel composition consisted of five men and seven women, aged 23–60 years.

*Panel training:* Panellists completed four training sessions (60 min each), covering identification, definition of descriptors and intensity scaling using a structured 10 cm scale (0 = absent; 10 = very intense). Panellists were trained to identify and rank the intensity of each of the following descriptors: “fresh fruit”, “dried fruit”, “spirit-like”, “oxidation”, “reduction”, “astringency”, “bitterness”, and “body”. Preparation protocols and concentrations are detailed in Table S3 of Supplementary Material. The terms “positive odour intensity” (POI), and “in-mouth balance” were also defined. POI refers to the amount of positive odour perceived orthonasally, evaluated by comparing with a reference built in the lab that has an average intensity (5 in a 0–10 scale). The red-wine like POI reference was prepared mixing the aroma compounds reported in de-la-Fuente-Blanco, Arias-Pérez, Escudero, Sáenz-Navajas, and Ferreira (2024). In-mouth balance was defined as: ‘*Linked to the concept of edge. An edge would be the inappropriate intensity of acidity, astringency or bitterness, either due to excess or deficiency*’. There are no references for this attribute, so its use was based on its definition and on the definition of the points on the scale (0: completely unbalanced with several significant edges or one very significant edge; 2: unbalanced with several edges or one significant edge; 5: balanced with some slight edge; 7: balanced with no significant edges; 10: very balanced with no edges).

In the last training session, participants were presented with 12 wine samples similar to those in the study, including four repeated samples to evaluate repeatability and consistency. To confirm panel performance, two two-way ANOVAs were performed, involving sample and judge as fixed factors, and first-order interaction. For each attribute, when significant sample-by-judge effects were identified, a Principal Component Analysis (PCA) was calculated on a table encoded in a sample × judge matrix, in which each cell represented the intensity evaluated by one judge in a sample. The purpose of conducting this PCA was to assess any discrepancies in the judges' scoring. Judges who demonstrated significant scores on the first PCA dimension of all attributes were retained for further consideration; those who did not meet this criterion were not qualified.

*Sample evaluation:* Each of the 36 samples (12 wines × 3 conditions) were evaluated in duplicate over two days, with four sessions per day. Each session included nine samples (9 samples × 4 sessions × 2 days = 72 samples). Aroma was evaluated orthonasally for the next attributes: fresh fruit, dried fruit, oxidation, reduction, spirit-like aroma, and POI. For taste and mouthfeel attributes, astringency, bitterness, body and in-mouth balance were evaluated. All attributes were rated on a 0–10 scale described above.

*Data analysis:* For each sensory attribute, ANOVA with judge as random factor and treatment as fixed factor (control, oxidation or thermal treatment) was calculated.

#### 2.3.2 Chemical analysis

*Tannin activity.* Tannin activity was calculated as the specific enthalpy of interaction between tannins and a hydrophobic surface (polystyrene divinylbenzene HPLC column) as proposed by Revelette, Barak, and Kennedy (2014). Detailed information of the chromatographic conditions employed can be found in Ferrero-del-Teso et al. (2022).

*Voltammetry.* Linear sweep voltammograms were acquired between 0 and 1200 mV using a Nomasense Polyscan analyser (Nomacorc, Belgium) equipped with disposable screen-printed carbon electrodes and an Ag/AgCl reference electrode as previously described by Sáenz-Navajas et al. (2023). The first-order derivative of the measured current was calculated to facilitate visualisation and comparison of voltammetric differences among samples. Because the original voltammograms exhibited broad and overlapping anodic waves without well-resolved peaks, derivative processing was applied to enhance inflection regions and improve the identification of reproducible electrochemical transitions across wines. PCA with Varimax rotation was calculated using the first derivative of the 120 measurements acquired for each of the 36 wines as described in detail in Sáenz-Navajas et al. (2023). Components with eigenvalues >1 were retained for further interpretation according to the Kaiser criterion, while also considering the cumulative explained variance and the interpretability of the electrochemical regions represented by the components. Accordingly, five principal dimensions (D1–D5) were extracted from the PCA. To evaluate the effect of treatment, an analysis of variance (ANOVA) was performed on the 36 wine scores obtained for each dimension, considering treatment as a fixed factor. When significant effects were detected, Bonferroni-adjusted post hoc pairwise comparisons were applied to determine differences in voltammetric signals among the three treatments (Control, ATT, and COx). All statistical analyses were carried out using XLSTAT software (Addinsoft, version 2023.1.1).

#### 2.3.3 Classification model to predict oxidation susceptibility

*Classification of wines attending to wine susceptibility:* The response variable was defined as a binary variable based on the sensory parameter dQ = Q_(OX) – Q_(CT), which expresses the loss of quality induced by oxidative treatment: wines with dQ < −1 were considered susceptible and the remainder were considered non-susceptible. Wines were classified according to their oxidation susceptibility using a dQ threshold of −1. This threshold was selected because it represented a clear sensory decrease in quality relative to control wines, exceeding the average intra-panel variability observed during sensory evaluation. The criterion was therefore intended to distinguish wines showing perceptible oxidative deterioration from those with limited sensory impact. It should be noted that this threshold was operational rather than absolute.

*Classification performance and internal validation of the nested PCA–LDA model:* two levels of the response variable (high or low oxidation susceptibility) were considered. The predictor variables were the rotated principal component analysis (PCA) dimensions calculated from the first derivative of the voltammetric signals. To avoid information leakage during model validation, a fully nested leave-one-out cross-validation (LOOCV) strategy was implemented. Within each LOOCV iteration, PCA with Varimax rotation was recalculated exclusively using the training wines. Consequently, twelve independent PCA models were generated throughout the cross-validation procedure. Because the rotated PCA dimensions varied slightly between iterations, the PCA decomposition was considered fold-specific and used exclusively for classification purposes. The excluded wine was subsequently projected onto the PCA space obtained from the corresponding training set. For this purpose, the original voltammetric variables of the excluded wine were standardised using the mean and standard deviation of the training wines from the corresponding fold. The standardised variables were then projected into PCA coordinates using the Varimax rotation transformation coefficients provided by each PCA model.

Linear discriminant analysis (LDA) was subsequently calculated using the PCA scores obtained for the training wines. To reduce model complexity and minimise overfitting risk associated with the limited sample size, only parsimonious two-dimensional LDA models were considered. All possible combinations of two PCA dimensions were evaluated independently within each fold, and the combination providing the highest classification performance for the training wines was retained for classification of the excluded wine. The discriminant function generated from the selected LDA model was then applied to the projected PCA coordinates of the excluded wine in order to predict its oxidation susceptibility class. This procedure was repeated iteratively until every wine had been used once as independent test sample.

*External validation of the model:* to further validate the model and confirm that the observed classification performance was not arose by chance, a permutation test was additionally performed. Oxidation susceptibility labels were randomly reassigned while maintaining the original PCA scores and fold structure. The complete nested PCA–LDA workflow, including dimensionality selection and classification, was repeated for 100 permuted datasets, and the resulting distribution of accuracies was compared with the observed classification accuracy. The proportion of permuted models yielding accuracies equal or greater than the real model was calculated.

*Identification of important variables:* to further evaluate the electrochemical regions contributing to oxidation susceptibility discrimination, the importance of the voltammetric regions associated with the nested PCA–LDA models was additionally assessed. Because PCA dimensions varied slightly between LOOCV folds due to the independent recalculation of PCA with Varimax rotation, interpretation was performed at the level of the original voltammetric regions rather than at the level of the fold-specific dimensions themselves. For each fold-specific model, the voltammetric regions contributing to the selected PCA dimensions were identified from the corresponding rotated factor loadings. A normalised importance index was calculated for each voltammetric region by combining its contribution (%) to the PCA dimensions selected in the optimal fold-specific LDA models with the absolute value of the corresponding LDA coefficients. The resulting normalised contributions were aggregated across all LOOCV iterations and normalised to the total importance, yielding the normalised contribution of each electrochemical region to the overall classification performance.

All statistical analyses were performed using XLSTAT (2023 1.1.) and R (version R 4.5.2).

## 3 Results

### 3.1 Sensory effects of the oxidation treatment

#### 3.1.1 Projective mapping with wine experts: holistic evaluation

**Fig. 1:**
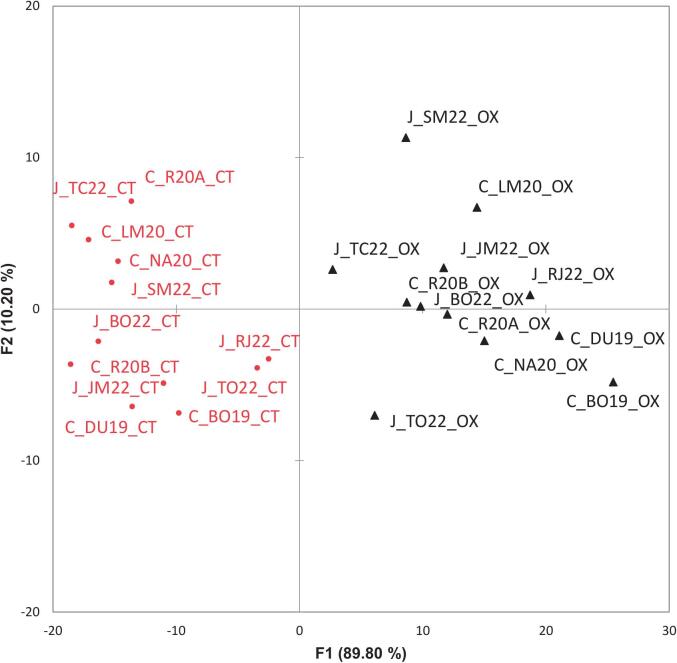
Consensual map of the projective mapping performed by 12 wine experts with 24 wine samples: 12 control (in red) and 12 submitted to oxidation conditions (in black). (For interpretation of the references to colour in this figure legend, the reader is referred to the web version of this article.)

Fig. 1 shows the consensual map derived from the projective mapping performed by the panel of experts with the 12 samples submitted to oxidation treatment and their control counterparts. The first dimension, accounting for approximately 90% of the original variance, evidently differentiates between control and treated samples. This underscores the pivotal effect of the oxidation treatment on the resulting samples. This dimension exhibits a clear negative correlation with perceived quality (r = – 0.93; F = 138.5, *p* < 0.0001) and positive with the REDOX score (*r* = 0.81; F = 41.61, *p* < 0.0001), as evaluated by the panel of experts. The more on the right part of the plane, where the treated samples are projected, the lower the perceived quality and the more oxidised the wines are. Dimension 1 is a clear sign of quality decline and oxidation. However, it is also seen that not all samples are affected in the same way by the treatment. For example, samples C_BO19 and J_TC22 show the biggest and smallest distances between the control and treated samples. This finding serves to support H1 confirming that wines subjected to identical oxidative conditions exhibited markedly different levels of sensory deterioration and quality loss, depending on their initial composition.

#### 3.1.2 Descriptive analysis with a trained panel: analytical evaluation

Table 1 illustrates the mean values for the sensory attributes evaluated by the trained panel for both the control and treated samples. The oxidation process resulted in substantial alterations to the sensory characteristics of the samples. These alterations manifested as an overall decline in the intensity of positive aromas, POI, (from 5.1 to 4.0), a significant decrease in fresh fruit aromas (from 4.1 to 2.7), and an increase in dried fruit aromas (from 3.2 to 4.7). Additionally, oxidation aromas (from 2.1 to 4.0) and spirit-like aromas (from 4.0 to 4.8) were also observed to increase with the treatment. The bitterness level exhibited a notable increase ranging from 4.6 to 5.5, followed by a significant rise in astringency from 5.8 to 6.4. All this resulting in a decrease in the in-mouth balance with the treatment (from 5.2 to 4.4).

**Table 1:** Average (±error: sd/√n; sd: standard deviation; n= number of subjects performing the sensory evaluation) of control samples and the corresponding samples submitted to oxidation. Significance (*p*) and ANOVA F-statistic (F) of the treatment effect on the 10 descriptors scored by the trained panel and two global terms (REDOX and Quality) evaluated by a panel of experts.

	Control	COx	F	*p*
POI	**5.1 ± 0.2**	4.0 ± 0.3	21.90	***
Fresh fruit	**4.1 ± 0.4**	2.7 ± 0.3	13.96	**
Dried fruit	3.2 ± 0.2	**4.7 ± 0.3**	23.38	***
Reduction	0.7 ± 0.2	0.6 ± 0.3	0.52	ns
Oxidation	2.1 ± 0.3	**4.0 ± 0.2**	18.67	***
Spirit-like	4.0 ± 0.2	**4.8 ± 0.2**	9.39	**
Bitterness	4.6 ± 0.2	**5.5 ± 0.2**	16.60	***
Astringency	5.8 ± 0.3	**6.4 ± 0.3**	4.40	*
Body	**5.2 ± 0.1**	4.9 ± 0.1	5.12	*
In-mouth balance	**5.2 ± 0.3**	4.4 ± 0.2	8.12	**
REDOX	0.3 ± 0.1	**1.8 ± 0.3**	48.00	***
Quality	**5.5 ± 0.2**	4.3 ± 0.2	23.34	***

Signification codes (*p*): <0.05*; <0.01**; <0.001***.

The effect of the COx on the aroma profile is consistent with the formation of Strecker aldehydes, acetals, and sotolon as key markers of oxidative deterioration reported in previous papers (Aragón-Capone, De-La-Fuente-Blanco, et al., 2025). Furthermore, our study expands the sensory evaluation to include in-mouth parameters (e.g., bitterness, astringency, and overall balance), facets not addressed in the earlier work, thereby providing a broader understanding of oxidative impacts on wine sensory quality. Regarding the increase in bitterness and specially astringency observed during wine oxidation are consistent with the highly significant increase in tannin activity (F = 37.53; *p* < 0.0001) from 2798 ± 577 J mol^−1^ to 4353 ± 663 J mol^−1^. According to the definition proposed by Revelette et al. (2014), tannin activity refers to the energetic capacity of tannins to interact with and precipitate salivary proteins, quantified as the enthalpy change associated with tannin–protein binding. This parameter reflects the structural features of tannins and their reactivity toward proteins, making it a relevant proxy for perceived astringency (Ferrero-del-Teso et al., 2022). This result can be attributed to the formation of quinones and to oxidative polymerisation processes affecting the phenolic fraction. The oxidation of flavanols and hydroxycinnamic acids leads to highly reactive quinones that participate in condensation, coupling and polymerisation reactions, generating larger and structurally more rigid phenolic species yielding higher tannin activity (Nguyen & Waterhouse, 2021; Oliveira et al., 2011). These oxidation products contribute directly to bitterness and simultaneously enhance astringency by increasing the affinity of tannins for salivary proteins, thereby promoting precipitation and friction-related sensory effects (Soares, Brandão, Mateus, & de Freitas, 2017).

### 3.2 Sensory effects of the thermal treatment

#### 3.2.1 Projective mapping with wine experts: holistic evaluation

**Fig. 2:**
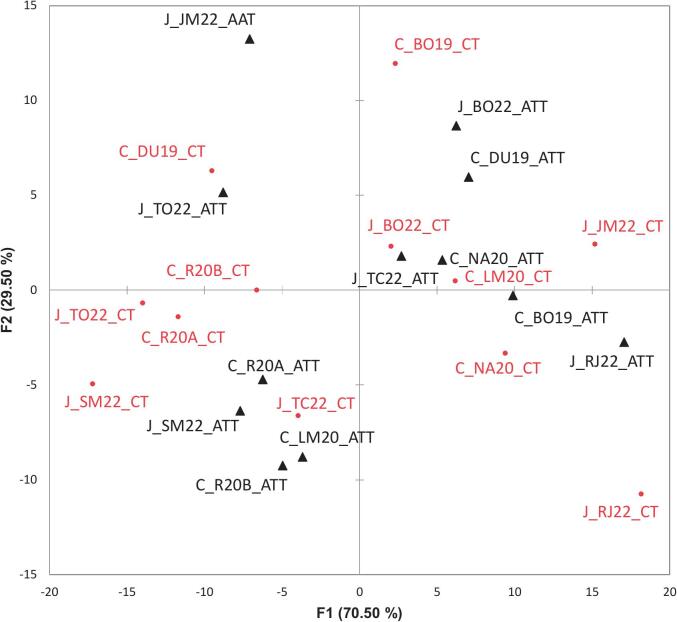
Consensual map of the projective mapping performed by 12 wine experts with 24 wine samples: 12 control (in red) and 12 submitted to thermal treatment conditions (in black). (For interpretation of the references to colour in this figure legend, the reader is referred to the web version of this article.)

Fig. 2 displays the projection of the 24 samples that were subjected to projective mapping. The first dimension (F1) accounts for over 70% of the original variance. F1 is found to be significantly and positively correlated with perceived quality (*r* = 0.48; F = 6.48; *p* < 0.05). The samples positioned on the right side of the plot are perceived to be of a higher quality than those on the left. However, no effect of the treatment on the positioning of the samples on F1 can be observed. Even if no salient sensory differences (i.e., positioning on F1) among samples associated with the treatment is observed, ANOVA calculated with the quality scores showed a discreet effect of the treatment on perceived quality (control = 5.5 vs thermal treatment = 5.0, Table 2).

**Table 2:** Average (±error: sd/√n; sd: standard deviation; n=number of subjects performing the sensory evaluation) of control samples and the same samples submitted to thermic treatment. Significance (P) of the effect of the treatment on 10 descriptors scored by the trained panel and two global terms (REDOX and Quality) evaluated by a panel of experts.

	Control	ATT	F	*p*
POI	5.1 ± 0.2	4.7 ± 0.2	1.77	ns
Fresh fruit	**4.1 ± 0.4**	3.3 ± 0.4	6.45	*
Dried fruit	3.2 ± 0.2	3.5 ± 0.4	1.00	ns
Reduction	0.7 ± 0.3	1.4 ± 0.5	4.23	ns
Oxidation	2.1 ± 0.2	2.1 ± 0.3	0.06	ns
Spirit-like	4.0 ± 0.2	4.4 ± 0.3	2.83	ns
Bitterness	**4.6 ± 0.2**	4.1 ± 0.2	5.49	*
Astringency	5.8 ± 0.3	5.7 ± 0.1	0.25	ns
Body	5.2 ± 0.1	5.4 ± 0.1	1.33	ns
In-mouth balance	5.2 ± 0.3	5.3 ± 0.2	0.17	ns
REDOX	0.3 ± 0.1	0.2 ± 0.3	0.22	ns
Quality	**5.5 ± 0.2**	5.0 ± 0.3	5.28	*

Signification codes (P): <0.05*; <0.01**; <0.001***.

The second dimension, accounting for approximately 30% of the original variance, exhibited a discreet, albeit non-significant, correlation with the sensory REDOX descriptor (*r* = 0.36; F = 3.24; *p* < 0.1, Table 2).

Overall, the projective mapping results indicate that the applied ATT treatment induced relatively moderate sensory changes, primarily reflected in small variations in perceived quality rather than in major restructuration of the sensory space. Although Dimension 1 was significantly associated with perceived quality, the positioning of samples along this axis did not reveal a consistent treatment effect across all wines. Consequently, these results highlight that the ATT treatment did not generate a sufficiently strong sensory effect to robustly test H1 based solely on sensory data.

#### 3.2.2 Descriptive analysis with a trained panel: analytical evaluation

Table 2 illustrates the mean values for the sensory descriptors as evaluated by the trained panel. In general, the application of thermal treatment under anoxic conditions resulted in negligible sensory effects on the samples as observed in the projective mapping task. However, a decline in both bitterness and the fresh fruit aroma was discernible, which could provide a rationale for the decrease in perceived quality as determined by the expert panel. It is noteworthy that the maximal score for the reduction attribute attained a value of 2.8 (on a 10-point scale) for a treated sample (J_RJ22_ET), resulting in a REDOX score determined by the expert panel of −0.6. It is important to note that the REDOX score ranged from −5 (reduced) to +5 oxidised; 0 indicated neither oxidised nor reduced status. This finding indicates that the thermal treatment, despite eliciting a decline in perceived quality (from 5.5 to 5.0), did not result in the generation of reductive aromas (REDOX and reductive scores of control samples did not exhibit significant divergence from treatment, Table 2). This outcome contradicts our initial H1 related to the ATT treatment.

The limited sensory changes observed after thermal treatment under anoxic conditions extend previous findings suggesting that heat exposure in the absence of oxygen does not necessarily result in marked perceptible sensory shifts. Aragón-Capone et al. (2026) reported modifications in volatile profiles after 4 weeks at 35 °C, although only limited potential sensory relevance was inferred using odour activity values (OAVs). Furthermore, kinetic analyses have shown temperature-dependent evolution of reduction-related compounds under anoxic storage (Ainsa-Zazurca, Ontañón, & Ferreira, 2025). These observations, together with our sensory data, suggest that thermal stress alone may not be sufficient to induce major aromatic perception changes under the conditions tested, and that oxygen availability likely plays a primary role in driving substantial sensory shifts in red wines. However, stronger thermal stress, either through longer exposure times or higher storage temperatures, may be required to determine the conditions at which sensory effects become perceptible.

### 3.3 Effect of oxidation and thermal treatments on the voltammetric signals

**Fig. 3:**
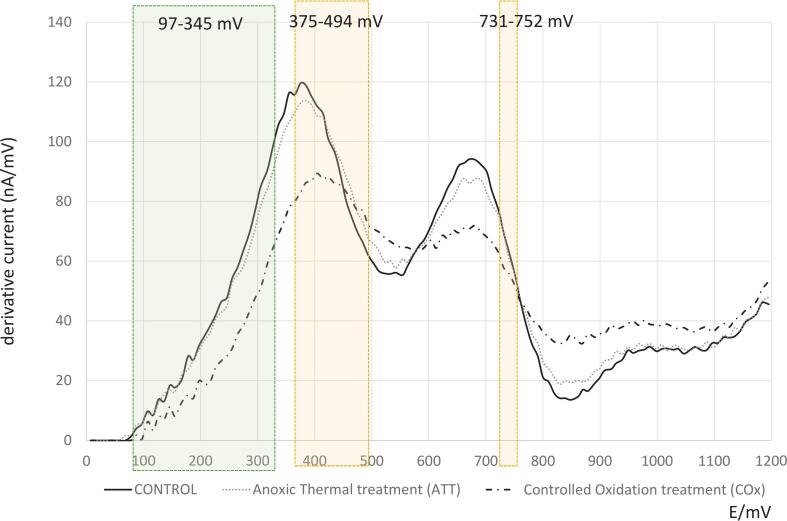
First derivative of the averaged (of 12 samples) voltammograms obtained for control, and samples submitted to control oxidation and anoxic thermal treatments. Regions marked in green (97–345 mV) and in yellow (375–494 mV; 731–752 mV) correspond to D4 and D2, respectively. (For interpretation of the references to colour in this figure legend, the reader is referred to the web version of this article.)

Fig. 3 shows the first derivative of the averaged (of 12 samples) voltammograms obtained for control, and samples submitted to oxidation and thermal treatments (original voltammograms are shown in Fig. S4 of Supplementary material). It shows that while the controlled oxidation treatment generated a clear effect on the voltammetric response of samples, the anoxic thermal treatment produced only a discreet effect. This result is well in line with the sensory results described in the previous section. The pronounced modification of voltammetric profiles induced by COx, in contrast to the limited effect of ATT, suggests that electrochemical signals capture oxidation-related chemical changes relevant to sensory deterioration, providing preliminary support for H2.

PCA with varimax rotation calculated on the first derivative of the voltammetric signals confirmed that there are five dimensions (D1-D5), which are linked to specific voltammogram regions (Table 3, and Table S4 Supplementary material), showing distinct and independent tendency along the voltammograms.

**Table 3:** Independent and non-correlated regions of the voltammogram linked to the five dimensions (D1-D5) obtained by PCA with varimax rotation, performed on the 36 wine samples (12 controls, 12 submitted to oxidation procedure and 12 to thermal treatment). The F-ratio and associated significance (*p*-value) correspond to the effect of treatment on each dimension (one-way ANOVA).

Dimension	Explained variance (%)	Regions of the voltammogram (mV)	F	*p*
D1	30.7	355–365; 504–573; 760–1087	2.93	ns
**D2**	**12.8**	**375–494; 731–752**	**4.21**	**<0.05**
D3	10.4	584–723	1.94	ns
**D4**	**22.9**	**97–346**	**7.99**	**<0.01**
D5	8.3	1097–1196	0.33	ns

Among the derivative-defined electrochemical regions identified in the voltammograms, the ranges 375–494 mV, 731–751 mV (D4), and particularly 97–346 Mv (D2) were significantly affected by treatment (*p* < 0.05; Table 3). Bonferroni-adjusted pairwise comparisons indicated that these differences were specifically driven by wines subjected to oxidative treatment, which exhibited significant decreases in the corresponding electrochemical transitions relative to control wines. In contrast, wines submitted to thermal treatment under anoxia did not differ significantly from controls in any region of the voltammogram. The localisation of these affected regions within the overall voltammetric profile is illustrated in Fig. 3.

As the ATT treatment was not able to induce relevant sensory and voltammetric changes related to the formation of reductive aromas, this treatment was not further considered and the following section is focused on the prediction of the oxidation susceptibility of wines induced by the COx treatment.

### 3.4 Prediction of the oxidation susceptibility of wines

This section specifically addresses H2, which postulates that initial voltammetric signals (referred to control samples) contain sufficient information to distinguish wines susceptible to oxidation−induced deterioration from those that are more resistant.

#### 3.4.1 Classification of wines based on oxidation susceptibility

**Fig. 4:**
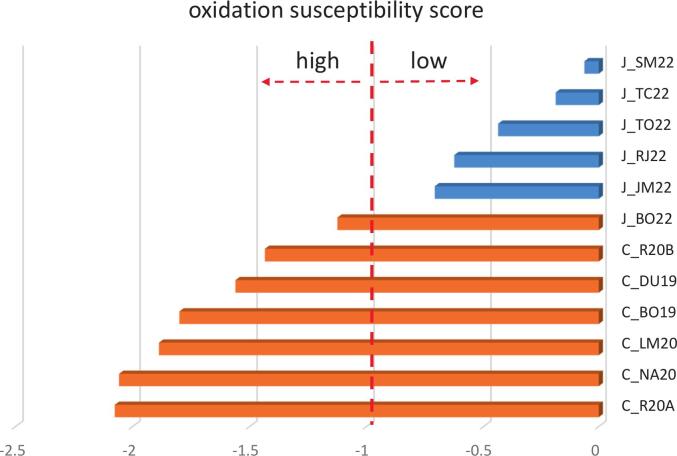
Oxidation susceptibility score (OSscore) for the 12 wines studied. Samples with OSscore > −1 are arbitrary classified as samples with low OSscore and samples with OSscore ≤ –1 are classified as samples with high OSscore.

The oxidation susceptibility (OSscore) was defined as the decrease in perceived quality (dQ) when applying the COx treatment (i.e. the difference between the quality score after treatment and control samples). Fig. 4 shows the graph with the oxidation susceptibility score for the 12 wines studied. Five of them, represented in blue, show low oxidation susceptibility (OSscore > −1), and seven wines, in orange, show high oxidation susceptibility (OSscore ≤ −1).

Notably, the wines exhibiting the lowest oxidation susceptibility were predominantly the young wines: the first five samples showed the lowest OSscores (low susceptibility), and the sixth—although slightly higher—still remained below the susceptibility levels observed in the aged wines, most of which had undergone oak maturation. This ageing−related pattern is coherent with the redox interpretation proposed in previous studies (Bueno et al., 2018) performed with young and aged commercial wines. Young wines generally retain higher concentrations of reactive polyphenols, small flavanols, and effective SO₂ buffering systems, which favour quinone scavenging and limit acetaldehyde accumulation through rapid nucleophilic addition and ethyl-bridge formation (Bueno-Aventín, Escudero, Fernández-Zurbano, & Ferreira, 2021; Nikolantonaki & Waterhouse, 2012). In the present study, tannin activity was significantly higher in the young wines than in the aged wines (F = 4.562; *p* = 0.044), supporting the presence of more structurally reactive and nucleophilic phenolic systems. Such reactivity may enhance oxidative protection through quinone scavenging, while also modulating the stability of polyfunctional mercaptans through tannin–thiol interactions under oxidative conditions, as previously suggested by Bueno-Aventín et al. (2021).

In contrast, aged wines, having undergone prolonged oxidative exposure during bottle and/or oak ageing, likely contain lower proportions of kinetically reactive phenolic structures together with reduced redox buffering capacity. This condition may favour the accumulation of oxidation−derived intermediates and consequently increase sensory oxidation susceptibility (Danilewicz, 2003; Razafindrabenja et al., 2026; Waterhouse & Miao, 2021). However, because wine age category and oak ageing were partially confounded in the present dataset, the respective contributions of bottle ageing and wood contact to oxidation susceptibility could not be fully disentangled.

#### 3.4.2 Classification performance of the nested PCA–LDA model

The nested PCA–LDA approach provided a consistent discrimination between wines exhibiting high and low oxidation susceptibility. Despite the fold-specific variability introduced by the independent recalculation of PCA with Varimax rotation, simplified two-dimensional discriminant models systematically provided the best compromise between model parsimony and classification performance. The nested leave-one-out cross-validation (LOOCV) procedure yielded an overall classification accuracy of 83.3%, with 10 of the 12 wines correctly classified according to their oxidation susceptibility category. These results suggest that the voltammetric information contained in the derivative-defined electrochemical profiles was associated with the sensory oxidative behaviour of the wines. Permutation testing further supported the non-random nature of the observed discrimination. Permutation testing (100 random permutations) showed that only one permuted dataset yielded a classification accuracy equal to or greater than that obtained with the true labels. This indicates that the observed classification performance was unlikely to arise from random assignment of oxidation susceptibility labels.

The model showed promising discrimination between wines with different oxidation susceptibility within the present dataset. The high classification accuracy obtained using voltammetric features provided preliminary evidence that initial electrochemical signatures can discriminate between oxidation−susceptible and non−susceptible wines. Notwithstanding, these results should be interpreted as exploratory and hypothesis−generating, requiring future validation using larger and independent wine datasets to confirm H2.

#### 3.4.3 Important variables of the model

**Fig. 5:**
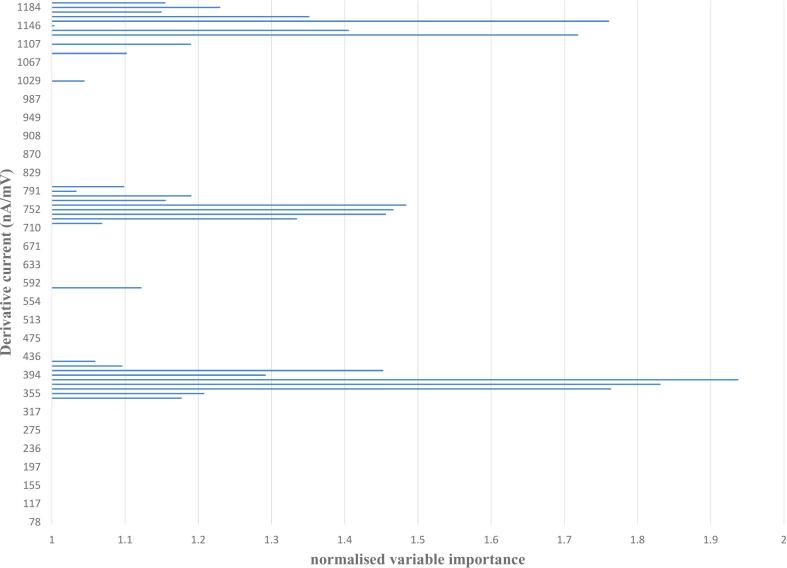
Normalised importance of the voltammetric regions contributing to oxidation susceptibility classification across the nested PCA–LDA models.

Fig. 5 shows the normalised importance of variables. Results revealed three electrochemical regions with relative importance values above the average (>1), indicating a consistent contribution to oxidation susceptibility discrimination across the nested PCA–LDA models (Fig. 5). These regions were located between 346 and 423 mV, 723–800 mV, and 1136–1196 mV. The 346–423 mV region showed the greatest contribution to the classification models, followed by the 1136–1196 mV region, whereas the 723–800 mV region displayed a smaller, although still above-average, contribution.

The chemical interpretation of the three most influential regions is consistent with both the voltammetric features observed in the original signals and established wine redox chemistry. The region located between 346 and 423 mV corresponds to the first shoulder observed in the original voltammograms (Fig. S4 of Supplementary Material) and to the first maximum in the first-derivative profiles (Fig. 3), representing the main region of electrochemical transition in the wine matrix. This potential domain is associated with the oxidation of the most readily oxidisable phenolic compounds, particularly o−diphenolic (catecholic) and galloylated structures such as flavan−3−ols and hydroxycinnamic derivatives (Kilmartin, Zou, & Waterhouse, 2001). Oxidation of these compounds generates highly reactive quinones and semiquinones involved in nucleophile depletion, oxidative cascades, colour evolution and aroma degradation (Bueno-Aventín et al., 2021; Danilewicz, 2003).

The second influential region (1136–1196 mV) corresponds to the third derivative maximum and therefore to another marked change in the voltammetric response occurring at higher potentials. The third important region (723–800 mV) approximately coincides with the second anodic wave observed in the original voltammograms (Fig. S4 of Supplementary Material) and with the second major peak in the derivative profiles (Fig. 3). This region is likely associated with less readily oxidisable phenolic compounds and structurally evolved oxidation products, reflecting a second major redox transition of the wine matrix. Although the precise electroactive species contributing to these regions cannot be unequivocally assigned, it likely reflects highly transformed phenolic constituents and other redox-active compounds requiring stronger oxidative conditions.

Importantly, the dimensions containing these regions consistently exhibited negative coefficients in the discriminant models. Because classification was based on the first derivative of the voltammetric response, these coefficients should be interpreted as reflecting differences in the magnitude of the electrochemical transitions rather than differences in absolute oxidation currents. Thus, wines exhibiting more pronounced changes in these key redox regions were generally less susceptible to sensory oxidation, suggesting a greater capacity of the wine matrix to undergo redox transformations and buffer oxidative stress. Conversely, wines displaying weaker transitions in these regions were more frequently classified as oxidation-susceptible. Overall, the results indicate that oxidation susceptibility is linked not only to the presence of oxidisable phenolic systems but also to the intensity and organisation of the electrochemical transitions occurring across the major redox domains of the wine matrix.

## 4 Conclusions

This study explored wine longevity through two complementary accelerated ageing approaches-controlled oxidation (COx) and anoxic thermal treatment (ATT)-combined with sensory evaluation, tannin activity and voltammetric analysis. Under the experimental conditions applied, COx consistently induced sensory changes associated with oxidative deterioration, including decreases in fruity aromas, increases in oxidative notes, and moderate but significant increases in bitterness and astringency. In contrast, ATT produced more limited sensory effects and did not lead to the development of clear reductive defects, suggesting that oxygen availability was the primary driver of the sensory changes observed within the timeframe of this study.

The increase in bitterness and astringency following COx treatment was accompanied by a significant rise in tannin activity, indicating enhanced tannin–protein interaction capacity. This observation is consistent with oxidative modifications of the phenolic fraction, such as quinone formation and polymerisation reactions, although the precise molecular mechanisms underlying these changes were not directly characterised and warrant further investigation.

Voltammetric measurements revealed treatment-dependent modifications of redox-active regions of the electrochemical signal, particularly under COx conditions. Multivariate analysis of these signals enabled discrimination between wines showing greater or lower susceptibility to oxidation-induced quality loss. Using a fully nested PCA–LDA framework, voltammetric profiles classified wines according to oxidation susceptibility with an overall accuracy higher than expected under random label assignment. Nevertheless, given the limited sample size, the model should be regarded as exploratory and hypothesis-generating rather than fully predictive.

The electrochemical regions most consistently associated with oxidation susceptibility were located between 346–423 mV, 723–800 mV and 1136–1196 mV. These derivative-defined regions correspond to major transitions in the voltammetric response and should be interpreted as operational electrochemical descriptors of the global wine redox behaviour rather than discrete oxidation processes of individual compounds.

A key limitation of the present study is the relatively small number of wines analysed, constrained by the high experimental workload associated with accelerated ageing treatments, sensory analysis and chemical characterisation. In addition, the wine set was dominated by Tempranillo-based wines, limiting varietal diversity. Furthermore, wine age category and oak ageing were partially confounded, preventing complete separation of their respective contributions to oxidation susceptibility. Future studies including larger, more diverse and compositionally balanced wine sets will therefore be necessary to validate the robustness and general applicability of the proposed models.

Overall, the findings indicate that voltammetric profiling captures chemically meaningful information related to wine redox behaviour and sensory evolution under oxidative stress. Although further validation is required, the results support the potential of electrochemical approaches as exploratory tools for assessing wine oxidation susceptibility and for supporting decision-making related to wine ageing and storage.

## CRediT authorship contribution statement

**Mónica Bueno:** Writing – review & editing, Methodology, Investigation, Conceptualization. **María-Pilar Sáenz-Navajas:** Writing – original draft, Visualization, Funding acquisition, Formal analysis, Data curation, Conceptualization. **Cristina Peña:** Project administration, Investigation, Conceptualization. **Ignacio Arias:** Investigation. **Carolina Castillo:** Investigation. **Purificación Fernández-Zurbano:** Writing – review & editing, Project administration, Funding acquisition, Conceptualization. **Arancha De la Fuente-Blanco:** Writing – review & editing, Investigation. **Chelo Ferreira:** Data curation, Writing – review & editing. **Ana Escudero:** Writing – review & editing, Project administration, Investigation, Funding acquisition, Conceptualization. **Vicente Ferreira:** Writing – review & editing, Project administration, Funding acquisition, Conceptualization.

## Declaration of competing interest

The authors declare that they have no known competing financial interests or personal relationships that could have appeared to influence the work reported in this paper.

## Data Availability

Data will be made available on request.

## Appendix A Supplementary data

Supplementary material. Additional tables, figures, methodological details, and supporting results related to this article.
