# Supplementary material for: Integrating sensory analysis and voltammetry to explore oxidative susceptibility in red wines
Source: Food Chem X. 2026 Jul 2;37:104163. doi: 10.1016/j.fochx.2026.104163 (PMC13356644; doi:10.1016/j.fochx.2026.104163)
Supplement: Supplementary file 1 — Supplementary material. Additional tables, figures, methodological details, and supporting results related to this article. [file mmc1.docx]

**SUPPLEMENTARY MATERIAL FOR**

**Integrating sensory analysis and voltammetry to explore oxidative susceptibility in red wines**

Mónica Bueno^1^, María-Pilar Sáenz-Navajas^2^*, Cristina Peña^1^, Ignacio Arias^2^, Carolina Castillo^2^, Purificación Fernández-Zurbano^2^, Arancha De la Fuente-Blanco^1^, Chelo Ferreira^3^, Ana Escudero^1^, Vicente Ferreira^1^

Corresponding author: mpsaenz@icvv.es

^1^Laboratorio de Análisis del Aroma y Enología (LAAE), Departament of Analytical Chemistry, Universidad de Zaragoza, Instituto Agroalimentario de Aragón (IA2) (UNIZAR-CITA) c/Pedro Cerbuna 12, 50009 Zaragoza, Spain

^2^Instituto de Ciencias de la Vid y del Vino (ICVV) (UR-CSIC-GR), Finca La Grajera, 26007 Logroño, La Rioja, Spain

^3^Instituto Universitario de Matemáticas y Aplicaciones (IUMA-–UNIZAR), Universidad de Zaragoza, c/ Pedro Cerbuna 12, 50009 Zaragoza, Spain

| **TABLE OF CONTENTS** | **Pages** |
| --- | --- |
| **Table S1.** List of 25 wine samples selected for screening and selection of the final list. | **S2** |
| **Table S2.** Conventional oenological parameters for the 12 selected samples. | **S3** |
| **Preliminary experiments** | **S4** |
| **Table S3.** List of references employed for panel training. | **S5** |
| **Figure S1.** Evolution of reduction, fresh fruit and astringency notes over time in the incubation of two wines (SED and MV) at 35°C under anoxic conditions. | **S6-S7** |
| **Figure S2.** Sensory evaluation of the fresh fruit and dried fruit notes in two wines (SED and MV) under seven conditions (Oxygen × Temperature). | **S7** |
| **Figure S3.** Sensory evaluation of the astringency note in two wines (PEB and MV) under four different temperature conditions (6, 22, 35, and 50 °C) at a fixed oxygen dose (0 mg/L, no added oxygen), | **S7** |
| **Figure S4.** Voltammograms of two wines (C_R20A in orange and J_JM22 in blue) for control (C), and samples submitted to control oxidation (CT) and anoxic thermal treatments (ATT) | **S8** |
| **Table S4.** The range of potential applied to wine samples (from 97-1196 V) and cos^2^ of the variables after rotation for the five dimensions | **S9-S11** |

**Table S1.** List of 25 wine samples initially selected for screening and selection of the final list of 12 wines (marked with an asterisk).

| **Code** | **Origin** | **Year** | **Type** | **Varieties** | **Alcohol content (%,v/v)** |
| --- | --- | --- | --- | --- | --- |
| ***C_LM20** | **La Mancha** | **2020** | **CRIANZA** | **Tempranillo Tinto/Cabernet Sauvignon** | **13.0%** |
| ***C_NA20** | **Navarra** | **2020** | **CRIANZA** | **Cabernet Sauvignon** | **14.0%** |
| C_NAb20 | Navarra | 2020 | CRIANZA | Tempranillo Tinto | 14.0% |
| ***C_R20A** | **Rioja** | **2020** | **CRIANZA** | **Tempranillo Tinto** | **13.5%** |
| C_Ri201 | Rioja | 2020 | CRIANZA | Tempranillo Tinto | 13.5% |
| C_Ri202 | Rioja | 2020 | CRIANZA | Tempranillo Tinto | 13.5% |
| C_Ri203 | Rioja | 2019 | CRIANZA | Tempranillo Tinto | 13.5% |
| C_Ri204 | Rioja | 2019 | CRIANZA | Tempranillo Tinto | 13.5% |
| ***C_R20B** | **Rioja** | **2020** | **CRIANZA** | **Tempranillo Tinto** | **14.0%** |
| C_RD191 | Ribera del Duero | 2019 | CRIANZA | Tinta del País | 14.5% |
| ***C_DU19** | **Ribera del Duero** | **2019** | **CRIANZA** | **Tinta del País** | **15.0%** |
| C_SM19 | Somontano | 2019 | CRIANZA | Coupage | 14.0% |
| ***C_BO19** | **Campo de Borja** | **2019** | **CRIANZA** | **Garnacha Tinta/Tempranillo Tinto** | **14.0%** |
| C_VAL19 | Valdepeñas | 2019 | CRIANZA | Tempranillo Tinto | 13.0% |
| J_RJ221 | Rioja | 2022 | YOUNG | Tempranillo Tinto | 13.0% |
| ***J_RJ22** | **Rioja** | **2022** | **YOUNG** | **Tempranillo** | **13.5%** |
| J_BO221 | Campo de Borja | 2022 | YOUNG | Garnacha Tinta | 14.0% |
| ***J_SM22** | **Somontano** | **2022** | **YOUNG** | **Merlot/Tempranillo Tinto** | **13.0%** |
| ***J_BO22** | **Campo de Borja** | **2022** | **YOUNG** | **Garnacha/Syrah** | **13.5%** |
| J_CAR22 | Cariñena | 2022 | YOUNG | Garnacha Tinta | 13.5% |
| ***J_TC_22** | **IGP Tierra Castilla** | **2022** | **YOUNG** | **Tempranillo Tinto/Cabernet Sauvignon** | **14.0%** |
| ***J_TO22** | **Toro** | **2022** | **YOUNG** | **Tinta Toro** | **14.0%** |
| J_CAT22 | Cataluña | 2022 | YOUNG | Garnacha Tinta | 13.0% |
| J_JM221 | Jumilla | 2022 | YOUNG | Monastrell/Syrah | 14.0% |
| ***J_JM22** | **Jumilla** | **2022** | **YOUNG** | **Monastrell** | **14.5%** |

**Table S2.** Conventional oenological parameters for the 12 selected samples.

| Code | SO_2_ (free)  (mg/L)^b^ | SO_2_ total  (mg/L)^b^ | Titratable acidity (g/L)^a^ | pH^a^ | TPI^c^ | Tannin activity  (-J/mol)^d^ | Total tannins  (mg/L)^d^ |
| --- | --- | --- | --- | --- | --- | --- | --- |
| C_LM20 | 27.2 ± 2.3 | 99.2 ± 2.3 | 4.7 ± 0.0 | 3.7 ± 0.0 | 64.0 ± 0.1 | 2452 ± 61 | 4468 ± 32 |
| C_NA20 | 12.8 ± 4.5 | 55.2 ± 14.7 | 4.8 ± 0.0 | 3.6 ± 0.0 | 53.2 ± 0.2 | 2172 ± 215 | 3629 ± 1 |
| C_R20A | 11.2 ± 4.5 | 80.8 ± 5.7 | 5.1 ± 0.0 | 3.5 ± 0.0 | 52.1 ± 3.7 | 2727 ± 140 | 3239 ± 67 |
| C_R20B | 16 ± 2.3 | 54.4 ± 2.3 | 4.6 ± 0.0 | 3.7 ± 0.0 | 57.1 ± 0.2 | 2372 ± 292 | 3986 ± 75 |
| C_DU19 | 6.4 ± 2.3 | 24.0 ± 2.3 | 5.3 ± 0.0 | 3.6 ± 0.0 | 74.3 ± 0.4 | 3465 ± 178 | 5345 ± 91 |
| C_BO19 | 21.6 ± 3.4 | 108.0 ± 1.1 | 5.4 ± 0.0 | 3.5 ± 0.0 | 62.9 ± 8.4 | 2109 ± 188 | 3654 ± 54 |
| J_RJ22 | 17.6 ± 0.0 | 31.2 ± 1.1 | 4.4 ± 0.0 | 3.7 ± 0.0 | 60.9 ± 0.1 | 3913 ± 194 | 4369 ± 2 |
| J_SM22 | 16.0 ± 2.3 | 52.8 ± 2.3 | 4.9 ± 0.0 | 3.7 ± 0.0 | 51.1 ± 0.3 | 2897 ± 166 | 3614 ± 59 |
| J_BO22 | 12.0 ± 5.7 | 68.0 ± 12.4 | 5.0 ± 0.0 | 3.5 ± 0.0 | 47.7 ± 0.0 | 2186 ± 108 | 3063 ± 31 |
| J_TC_22 | 22.4 ± 2.3 | 56.8 ± 5.7 | 4.7 ± 0.0 | 3.7 ± 0.0 | 72.3 ± 0.1 | 2983 ± 304 | 4283 ± 78 |
| J_TO22 | 23.2 ± 1.1 | 34.8 ± 1.7 | 4.9 ± 0.0 | 3.7 ± 0.0 | 57.1 ± 0.1 | 3417 ± 107 | 3970 ± 72 |
| J_JM22 | 19.2 ± 0.0 | 47.6 ± 1.7 | 5.2 ± 0.0 | 3.6 ± 0.0 | 55.3 ± 1.0 | 2880 ± 33 | 3930 ± 12 |
| ^a^Alcohol content, pH and titratable acidity (expressed as g/L of tartaric acid) were analysed by Infrared Spectrometry with Fourier Transformation with a WineScanTM FT 120 (FOSS®, Barcelona, Spain), which was previously calibrated with the official OIV methods.  ^b^For free and total sulphur dioxide determination, the aspiration/titration method recommended by the OIV was used (OIV, 2009).  ^c^Total polyphenol index (TPI) was estimated as absorbance at 280 nm of samples diluted 1:100 in deionised water was measured in 1-cm-quartz cuvettes (Ribéreau-Gayon, 1970).  ^d^Concentration and activity of tannins was estimated by a UHPLC-UV-Vis method following the method proposed by Revelette, Barak, and Kennedy (2014).  **References**  Revelette, M. R., Barak, J. A., & Kennedy, J. A. (2014). High-performance liquid chromatography determination of red wine tannin stickiness. *Journal of Agricultural and Food Chemistry*, 62(28), 6626–6631.  Ribéreau-Gayon, P. (1970). Le dosage des composes phénoliques totaux dans les vins rouges. *Chemist-Analyst*, 52, 627–631.  OIV (2009). OIV-MA-AS323-04A Sulphur dioxide. In Compendium of International Methods of Analysis; OIV: Paris, France, 2009. | | | | | | | |

**Preliminary experiments**

Wines used in the preliminary experiments were selected because they corresponded to different grape varieties and exhibited distinct total polyphenol index values (measured as absorbance at 280 nm), ensuring great diversity.

For the preliminary experiments, bottles were recorked following the same protocol applied in the central study. All wines were sealed with DIAM 30 cork closures (DIAM Bouchage, France) to standardise oxygen ingress. Prior to use, closures were kept under anoxic conditions for at least 48 h to minimise the contribution of their initial internal oxygen and were transported in double-sealed bags with activated carbon. Bottles assigned to anoxic treatments were resealed under vacuum (–0.25 bar) using a commercial bottling line (Bodegas y Viñedos Ilurce, La Rioja, Spain), whereas bottles for controlled oxidation were prepared by adjusting headspace volume. After bottling, the upper surface of each cork was sealed with a two-component epoxy adhesive to prevent oxygen ingress at the closure interface.

**i) Thermal stability tests: ATT.** Sensory analysis of the three wines incubated at 35°C under strict anoxia for 14, 10, 5, 2, and 0 weeks indicated significant differences in astringency for one wine, in dried fruit notes for two wines, and in fresh fruit and reduction notes for all three wines. Figure S1 shows the evolution over time of the fresh fruit, reduction and astringency notes for two wines. The decrease in fresh fruit notes was wine-dependent; however, a 10-week incubation period was considered sufficient to induce stable loss of fresh fruit character. From week 10 onwards, reduction notes were not significantly different from their highest values in either wine. In contrast, significant changes in astringency were observed in only one of the three wines, reaching a minimum at week 5 and partially recovering thereafter, while the other two wines showed no significant variations, highlighting a wine-dependent response that warrants further investigation.

**Table S3.** Reference standards used for panel training and preparation procedures for the different intensity levels (*absence, low, medium, a*nd *high*).

| **Descriptor** | **Formulae and concentrations of references** |
| --- | --- |
| **Aroma Descriptors (absence-low-medium-high)** | |
| **Fresh Fruit** | Young red wine showing outstanding fresh fruit aroma (Eguren Ugarte, DOCa Rioja, 2023).  *Absence*: 8% ethanol solution; *low*: dilution 1:2 of original wine:8% ethanol solution; *medium*: 1:1 wine dilution; *high*: original wine |
| **Dried Fruit** | Young red wine (Eguren Ugarte, DOCa Rioja) stored at 50 ºC during 7 and 14 days.  *Absence*: original wine; *low*: dilution 1:1 of original wine:wine at 50ºC 7 days; *medium*: wine at 50 ºC during 7 days; *high*: 1:1 dilution of wines stored at 50ºC during 7 and 14 days |
| **Spirit-like** | Grape marc (40º, El Afilador, Zamora Company, Spain) diluted in mineral water.  *Absence:* mineral water*; low*: dilution 1:10 (grape marc:water); *medium*: dilution 1:5; *high*: dilution 1:2 |
| **Oxidation** | Young white (experimental Viura wine) wine stored at 50 ºC during 14 days.  *Absence*: original wine; low: dilution 2:1 of original wine:wine at 50 ºC during 14; *medium*: dilution 1:1 of original wine:wine at 50 ºC during 14; *high*: wine stored at 50ºC during 14 days |
| **Reduction** | Sodium Sulfide anhydrous in distilled water (pH = 9.6 with NaOH). This solution is added to a young red wine (Eguren Ugarte, DOCa Rioja) in order to get the following concentrations: a*bsence*: original wine; *low*: 100 µg/L of H_2_S; *medium*: 200 µg/L; *high*: 400 µg/L |
| **Taste and mouthfeel Descriptors (absence-low- medium-high)** | |
| **Sweetness** | Table sugar (0–3–6–12 g/L) in mineral water |
| **Acidity** | Tartaric acid (Scharlab, Spain) (0–3.7–7.5–1.5 g/L) in mineral water |
| **Bitterness** | Quinine sulphate dihydrate (Scharlab, Spain) (0–6–12–25 mg/L) in mineral water |
| **Astringency** | Aluminum potassium sulfate dodecahydrate (Sigma-Aldrich, Madrid) (0–1–2–3 g/L) in mineral water |
| **Body** | Carboxymethyl cellulose (Sosa, Spain) (0–1–2–4 g/L) in synthetic wine |
| **Synthetic wine:** 5 g/L of tartaric acid; 8% (v/v) of ethanol; pH=3.5 (adjusted with NaOH)  **Mineral water:** Mineral water OROTANA, distributed by Acquajet, Castellón (Spain) | |

**Figure S1.** Evolution of reduction, fresh fruit and astringency notes over time in the incubation of two wines (SED and MV) at 35°C under anoxic conditions. Different letters indicate significant differences between treatments for each parameter within each wine according to Fisher’s LSD test (95% confidence level). Astringency results for MV are not shown, as no significant differences were observed over time.


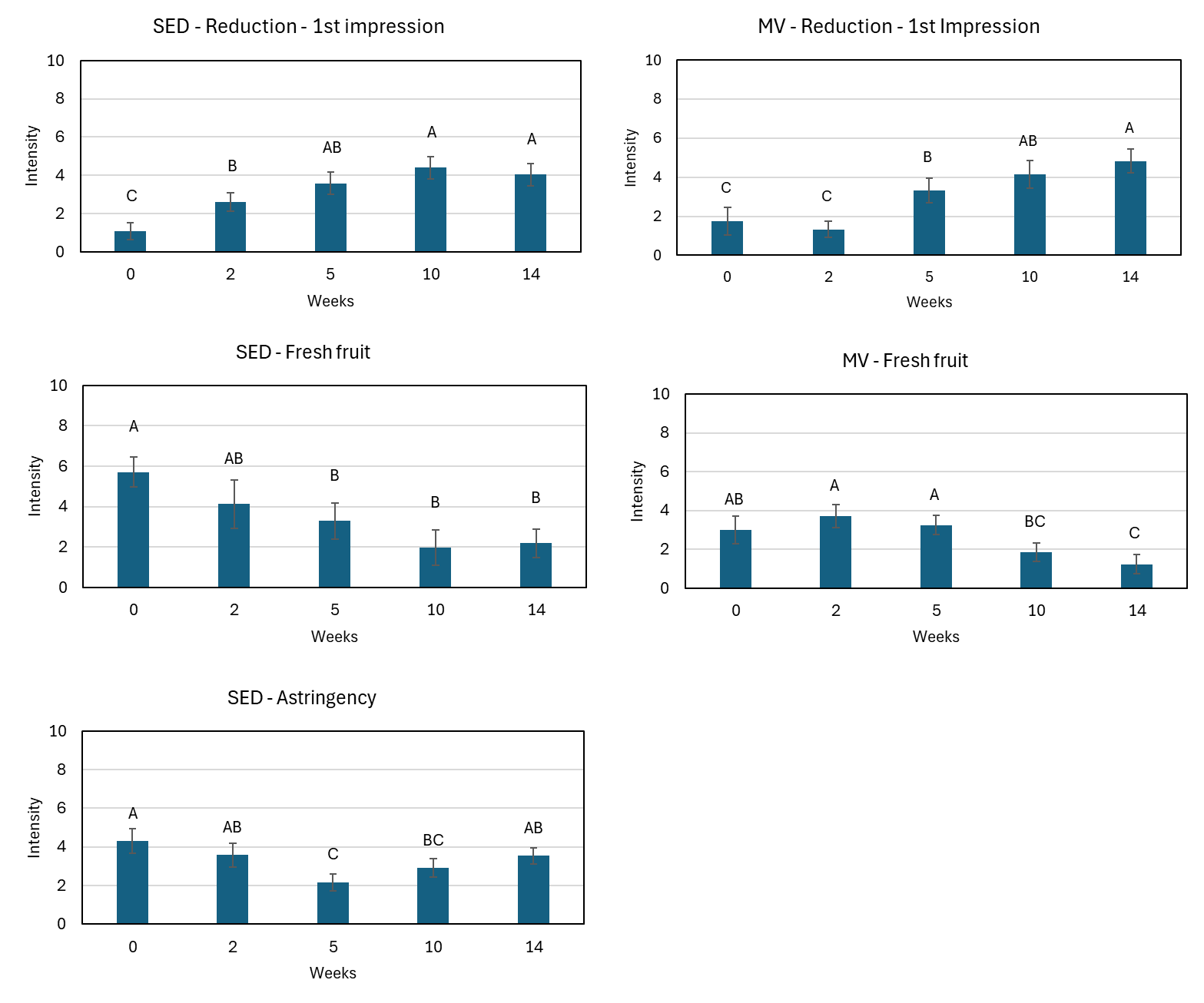


**ii) Controlled oxidation tests: COx.** Sensory analysis revealed that oxidative treatments applied for 10 weeks at different temperatures (6, 22, 35, and 50 °C) and initial oxygen doses (consisting of either no added oxygen or 35, 50, 65 mg/L over the stoichiometric amount to oxidise total SO_2_) produced significant differences in astringency in two wines and in oxidation, fresh fruit, and dried fruit notes in all four wines. Figure S2 presents the evaluation of the fresh fruit and dried fruit notes in two wines. It can be observed that an oxygen dose of 35 mg/L above the stoichiometric amount required to oxidise wine SO_2_ at 35 °C is sufficient to significantly decrease the fresh fruit notes and increase the dried fruit notes.

Figure S3 presents the evaluation of the astringency notes in the two wines for which this attribute showed significant differences. Changes in astringency were clearly wine-dependent. Under identical storage conditions (Oxygen_Temperature), MV showed a progressive increase in astringency, whereas PEB only exhibited changes under intermediate conditions. These differences may be related to compositional factors, such as protein–tannin interactions affecting protein tertiary structure, potentially involving modification of S-S disulfide bonds and formation of SH groups). Further experiments would be needed to determine the role of proteins in modulating astringency processes.

Therefore, the selected condition (35 mg/L O₂ over the stoichiometric SO_2_ at 35 °C) was considered suitable for generating measurable yet discriminant sensory deviations among wines.

**Figure S2.** Sensory evaluation of the fresh fruit and dried fruit notes in two wines (SED and MV) under seven conditions (Oxygen_Temperature). The fist number corresponds to oxygen addition (0 mg/L, no added oxygen; or 35, 50, and 65 mg/L oxygen above the stoichiometric amount required to oxidise wine total SO_2_), and the second number corresponds to storage temperatures (6, 22, 35, and 50 °C). Different letters indicate significant differences between treatments for each parameter within each wine according to Fisher’s LSD test (95% confidence level).


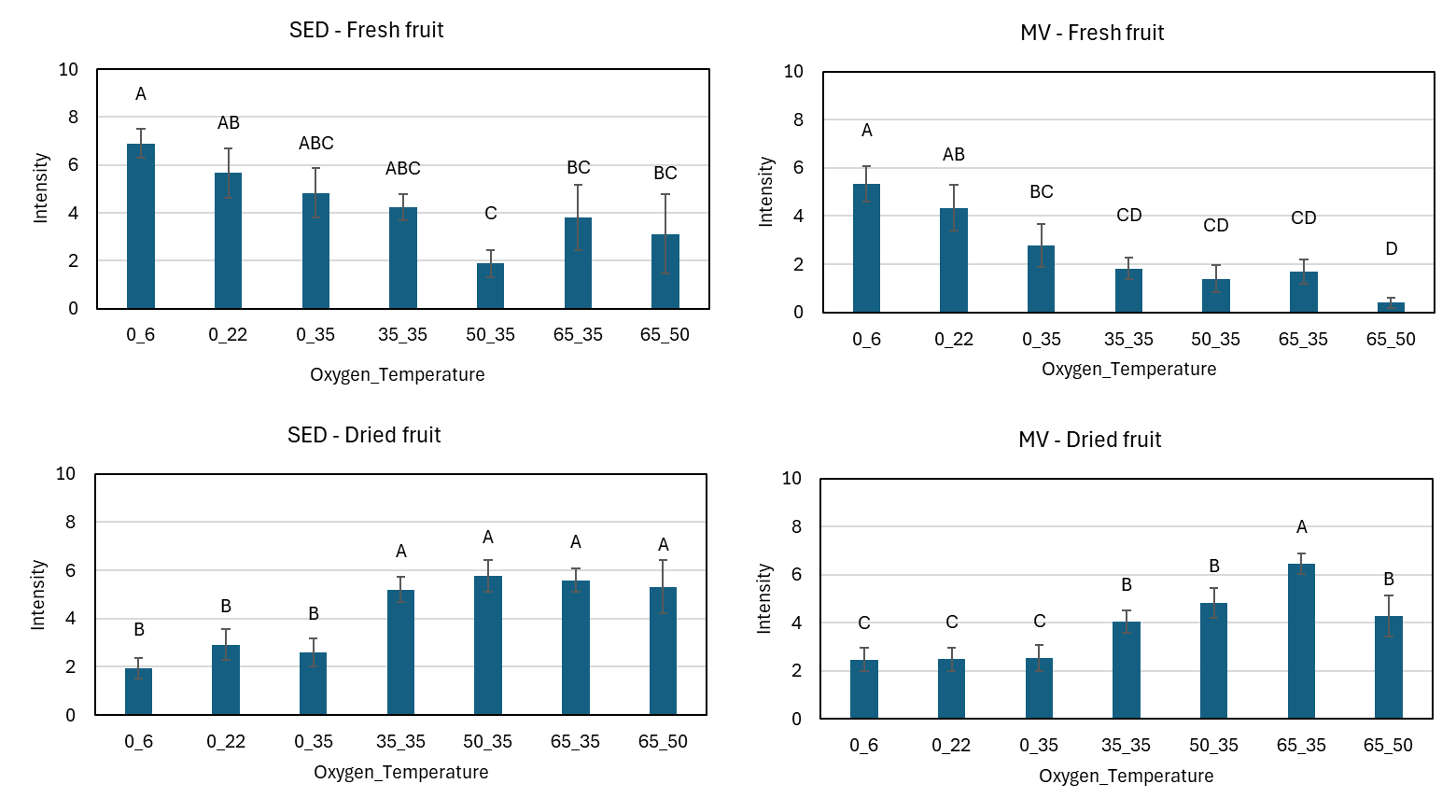


**Figure S3.** Sensory evaluation of the astringency note in two wines (PEB and MV) under four different temperature conditions (6, 22, 35, and 50 °C) at a fixed oxygen dose (0 mg/L, no added oxygen). Treatments are labelled as Oxygen_Temperature, where the fist number corresponds to oxygen dose and the second number corresponds to storage temperatures. Different letters indicate significant differences between treatments for each parameter within each wine according to Fisher’s LSD test (95% confidence level).


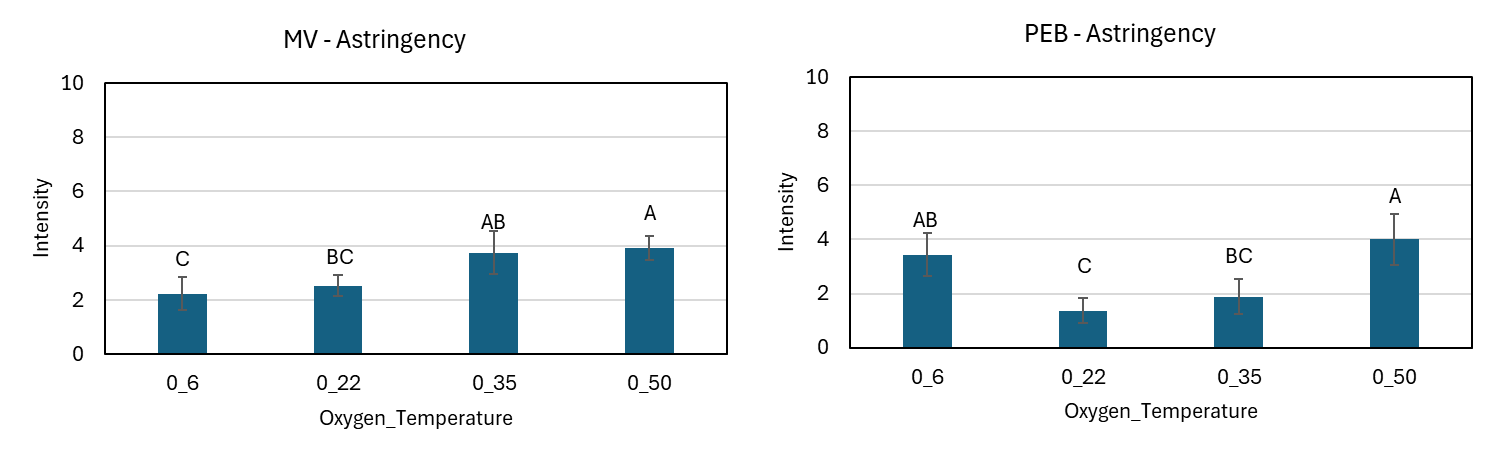


**Figure S4.** Voltammograms of two wines (C_R20A in orange and J_JM22 in blue) for control (C), and samples submitted to control oxidation (CT) and anoxic thermal treatments (ATT).

**
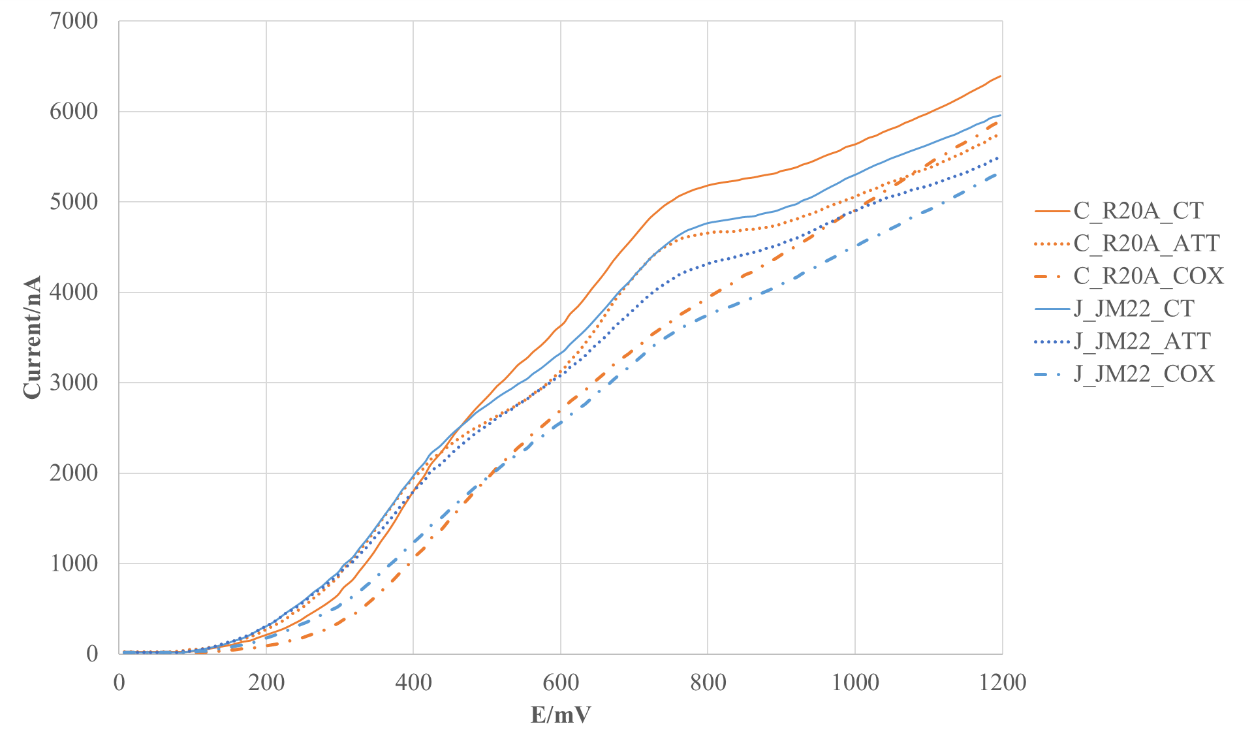
**

**Table S4.** The range of potential applied to wine samples (from 97-1196 mV) and cos^2^ of the variables after rotation for the five dimensions (D1-D5) identified by means of PCA with rotation applied to the 36 wine samples of the study (12 control, 12 submitted to oxidation and 12 to thermal treatments). Data marked in bold are significantly correlated with the dimension.

| **Energy (mV)** | **D1** | **D2** | **D3** | **D4** | **D5** |
| --- | --- | --- | --- | --- | --- |
| 97V | 0.008 | 0.011 | 0.001 | **0.616** | 0.034 |
| 107V | 0.011 | 0.031 | 0.077 | **0.330** | 0.020 |
| 117V | 0.037 | 0.026 | 0.135 | **0.477** | 0.005 |
| 126V | 0.001 | 0.018 | 0.003 | **0.550** | 0.000 |
| 136V | 0.029 | 0.048 | 0.041 | **0.513** | 0.037 |
| 146V | 0.046 | 0.028 | 0.061 | **0.538** | 0.000 |
| 155V | 0.050 | 0.020 | 0.019 | **0.569** | 0.083 |
| 167V | 0.014 | 0.034 | 0.042 | **0.617** | 0.027 |
| 178V | 0.109 | 0.043 | 0.026 | **0.518** | 0.017 |
| 187V | 0.061 | 0.047 | 0.022 | **0.691** | 0.043 |
| 197V | 0.043 | 0.039 | 0.062 | **0.652** | 0.091 |
| 207V | 0.117 | 0.002 | 0.002 | **0.694** | 0.012 |
| 217V | 0.074 | 0.032 | 0.036 | **0.701** | 0.096 |
| 226V | 0.138 | 0.009 | 0.050 | **0.663** | 0.031 |
| 236V | 0.180 | 0.005 | 0.026 | **0.696** | 0.027 |
| 246V | 0.136 | 0.004 | 0.006 | **0.730** | 0.030 |
| 255V | 0.273 | 0.002 | 0.071 | **0.574** | 0.021 |
| 265V | 0.243 | 0.011 | 0.021 | **0.618** | 0.043 |
| 275V | 0.343 | 0.001 | 0.046 | **0.526** | 0.015 |
| 286V | 0.310 | 0.000 | 0.008 | **0.601** | 0.010 |
| 296V | **0.451** | 0.000 | 0.037 | 0.424 | 0.021 |
| 305V | 0.346 | 0.002 | 0.036 | **0.555** | 0.001 |
| 317V | 0.445 | 0.013 | 0.030 | **0.449** | 0.004 |
| 326V | 0.329 | 0.052 | 0.044 | **0.476** | 0.011 |
| 336V | **0.442** | 0.035 | 0.032 | 0.407 | 0.008 |
| 346V | **0.394** | 0.096 | 0.022 | 0.390 | 0.014 |
| 355V | **0.373** | 0.147 | 0.058 | 0.338 | 0.013 |
| 365V | **0.402** | 0.257 | 0.012 | 0.277 | 0.020 |
| 375V | 0.308 | **0.345** | 0.045 | 0.241 | 0.015 |
| 384V | 0.263 | **0.465** | 0.033 | 0.187 | 0.020 |
| 394V | 0.191 | **0.681** | 0.014 | 0.057 | 0.005 |
| 404V | 0.047 | **0.853** | 0.009 | 0.011 | 0.032 |
| 415V | 0.023 | **0.912** | 0.014 | 0.001 | 0.007 |
| 423V | 0.004 | **0.922** | 0.000 | 0.012 | 0.014 |
| 436V | 0.014 | **0.856** | 0.011 | 0.062 | 0.004 |
| 446V | 0.107 | **0.756** | 0.001 | 0.081 | 0.001 |
| 455V | 0.119 | **0.645** | 0.003 | 0.161 | 0.000 |
| 465V | 0.213 | **0.573** | 0.000 | 0.158 | 0.000 |
| 475V | 0.225 | **0.489** | 0.001 | 0.206 | 0.003 |
| 484V | 0.326 | **0.440** | 0.004 | 0.197 | 0.000 |
| 494V | 0.341 | **0.390** | 0.000 | 0.221 | 0.008 |
| 504V | **0.396** | 0.303 | 0.000 | 0.217 | 0.010 |
| 513V | **0.421** | 0.297 | 0.000 | 0.235 | 0.003 |
| 523V | **0.478** | 0.208 | 0.000 | 0.238 | 0.007 |
| 533V | **0.440** | 0.234 | 0.004 | 0.220 | 0.014 |
| 542V | **0.449** | 0.165 | 0.013 | 0.238 | 0.033 |
| 554V | **0.496** | 0.137 | 0.010 | 0.203 | 0.020 |
| 562V | **0.498** | 0.113 | 0.047 | 0.207 | 0.022 |
| 573V | **0.353** | 0.128 | 0.200 | 0.049 | 0.015 |
| 584V | 0.173 | 0.126 | **0.332** | 0.027 | 0.031 |
| 592V | 0.073 | 0.038 | **0.661** | 0.026 | 0.017 |
| 604V | 0.006 | 0.002 | **0.703** | 0.000 | 0.005 |
| 613V | 0.031 | 0.023 | **0.731** | 0.071 | 0.000 |
| 623V | 0.089 | 0.014 | **0.715** | 0.093 | 0.002 |
| 633V | 0.132 | 0.001 | **0.647** | 0.128 | 0.004 |
| 642V | 0.224 | 0.003 | **0.548** | 0.120 | 0.028 |
| 652V | 0.198 | 0.006 | **0.507** | 0.241 | 0.011 |
| 662V | 0.245 | 0.000 | **0.532** | 0.147 | 0.020 |
| 671V | 0.185 | 0.001 | **0.574** | 0.187 | 0.019 |
| 681V | 0.197 | 0.000 | **0.468** | 0.216 | 0.069 |
| 692V | 0.202 | 0.021 | **0.493** | 0.167 | 0.042 |
| 702V | 0.026 | 0.059 | **0.515** | 0.192 | 0.136 |
| 710V | 0.041 | 0.106 | **0.522** | 0.119 | 0.116 |
| 723V | 0.000 | 0.210 | **0.359** | 0.094 | 0.181 |
| 731V | 0.016 | **0.341** | 0.273 | 0.053 | 0.173 |
| 741V | 0.092 | **0.305** | 0.246 | 0.001 | 0.183 |
| 752V | 0.240 | **0.398** | 0.058 | 0.009 | 0.188 |
| 760V | **0.349** | 0.292 | 0.045 | 0.026 | 0.148 |
| 771V | **0.422** | 0.302 | 0.000 | 0.125 | 0.042 |
| 781V | **0.422** | 0.284 | 0.004 | 0.170 | 0.035 |
| 791V | **0.488** | 0.186 | 0.027 | 0.197 | 0.004 |
| 800V | **0.445** | 0.159 | 0.037 | 0.273 | 0.004 |
| 810V | **0.560** | 0.076 | 0.014 | 0.266 | 0.002 |
| 820V | **0.522** | 0.062 | 0.063 | 0.273 | 0.001 |
| 829V | **0.533** | 0.054 | 0.035 | 0.311 | 0.003 |
| 839V | **0.555** | 0.016 | 0.065 | 0.275 | 0.007 |
| 849V | **0.540** | 0.007 | 0.057 | 0.327 | 0.009 |
| 862V | **0.494** | 0.021 | 0.036 | 0.307 | 0.004 |
| 870V | **0.611** | 0.013 | 0.057 | 0.225 | 0.024 |
| 879V | **0.539** | 0.000 | 0.075 | 0.280 | 0.028 |
| 891V | **0.609** | 0.001 | 0.050 | 0.198 | 0.019 |
| 899V | **0.674** | 0.001 | 0.029 | 0.154 | 0.064 |
| 908V | **0.688** | 0.001 | 0.083 | 0.075 | 0.021 |
| 920V | **0.632** | 0.000 | 0.081 | 0.119 | 0.026 |
| 928V | **0.727** | 0.002 | 0.053 | 0.056 | 0.069 |
| 939V | **0.736** | 0.009 | 0.033 | 0.022 | 0.040 |
| 949V | **0.837** | 0.000 | 0.055 | 0.008 | 0.016 |
| 958V | **0.759** | 0.001 | 0.062 | 0.018 | 0.057 |
| 968V | **0.854** | 0.001 | 0.010 | 0.005 | 0.019 |
| 978V | **0.876** | 0.002 | 0.028 | 0.019 | 0.017 |
| 987V | **0.830** | 0.021 | 0.017 | 0.022 | 0.008 |
| 999V | **0.770** | 0.002 | 0.049 | 0.006 | 0.047 |
| 1008V | **0.733** | 0.025 | 0.004 | 0.052 | 0.020 |
| 1017V | **0.734** | 0.012 | 0.007 | 0.046 | 0.094 |
| 1029V | **0.691** | 0.013 | 0.006 | 0.055 | 0.051 |
| 1037V | **0.545** | 0.070 | 0.002 | 0.071 | 0.055 |
| 1047V | **0.487** | 0.017 | 0.000 | 0.220 | 0.083 |
| 1058V | **0.524** | 0.005 | 0.031 | 0.130 | 0.097 |
| 1067V | **0.300** | 0.078 | 0.001 | 0.218 | 0.111 |
| 1078V | **0.382** | 0.000 | 0.013 | 0.191 | 0.125 |
| 1087V | **0.449** | 0.002 | 0.000 | 0.049 | 0.269 |
| 1097V | 0.162 | 0.023 | 0.038 | 0.173 | **0.272** |
| 1107V | 0.306 | 0.002 | 0.003 | 0.057 | **0.388** |
| 1117V | 0.047 | 0.003 | 0.005 | 0.187 | **0.383** |
| 1126V | 0.233 | 0.019 | 0.043 | 0.036 | **0.403** |
| 1136V | 0.131 | 0.000 | 0.006 | 0.101 | **0.445** |
| 1146V | 0.101 | 0.002 | 0.002 | 0.000 | **0.728** |
| 1155V | 0.009 | 0.014 | 0.009 | 0.096 | **0.652** |
| 1167V | 0.023 | 0.024 | 0.007 | 0.024 | **0.604** |
| 1175V | 0.032 | 0.002 | 0.006 | 0.020 | **0.571** |
| 1184V | 0.001 | 0.011 | 0.062 | 0.007 | **0.629** |
| 1196V | 0.044 | 0.076 | 0.022 | 0.003 | **0.594** |
